# Higher level domain specific skills in mathematics; The relationship between algebra, geometry, executive function skills and mathematics achievement

**DOI:** 10.1371/journal.pone.0291796

**Published:** 2023-11-06

**Authors:** Jayne Spiller, Sarah Clayton, Lucy Cragg, Samantha Johnson, Victoria Simms, Camilla Gilmore

**Affiliations:** 1 Centre for Mathematical Cognition, Loughborough University, Loughborough, United Kingdom; 2 School of Psychology and Vision Sciences, University of Leicester, Leicester, United Kingdom; 3 Department of Health Sciences, University of Leicester, Leicester, United Kingdom; 4 School of Psychology, University of Nottingham, Nottingham, United Kingdom; 5 School of Psychology, Ulster University, Coleraine, United Kingdom; The University of Adelaide, AUSTRALIA

## Abstract

Algebra and geometry are important components of mathematics that are often considered gatekeepers for future success. However, most studies that have researched the cognitive skills required for success in mathematics have only considered the domain of arithmetic. We extended models of mathematical skills to consider how executive function skills play both a direct role in secondary-school-level mathematical achievement as well as an indirect role via algebra and geometry, alongside arithmetic. We found that verbal and visuospatial working memory were indirectly associated with mathematical achievement via number fact knowledge, calculation skills, algebra and geometry. Inhibition was also indirectly associated with mathematical achievement via number fact knowledge and calculation skills. These findings highlight that there are multiple mechanisms by which executive function skills may be involved in mathematics outcomes. Therefore, using specific measures of mathematical processes as well as context-rich assessments of mathematical achievement is important to understand these mechanisms.

## 1. Introduction

Learning mathematics is important. Individuals with poor mathematics skills at age 21 are twice as likely to be unemployed at age 30 compared with individuals with sufficient mathematical skills [1]. It is therefore crucial to understand what skills contribute to success in mathematics. Recently, considerable attention has been paid to the role of domain-general skills, and specifically executive function skills, in explaining individual differences in mathematics outcomes.

**Fig 1:**
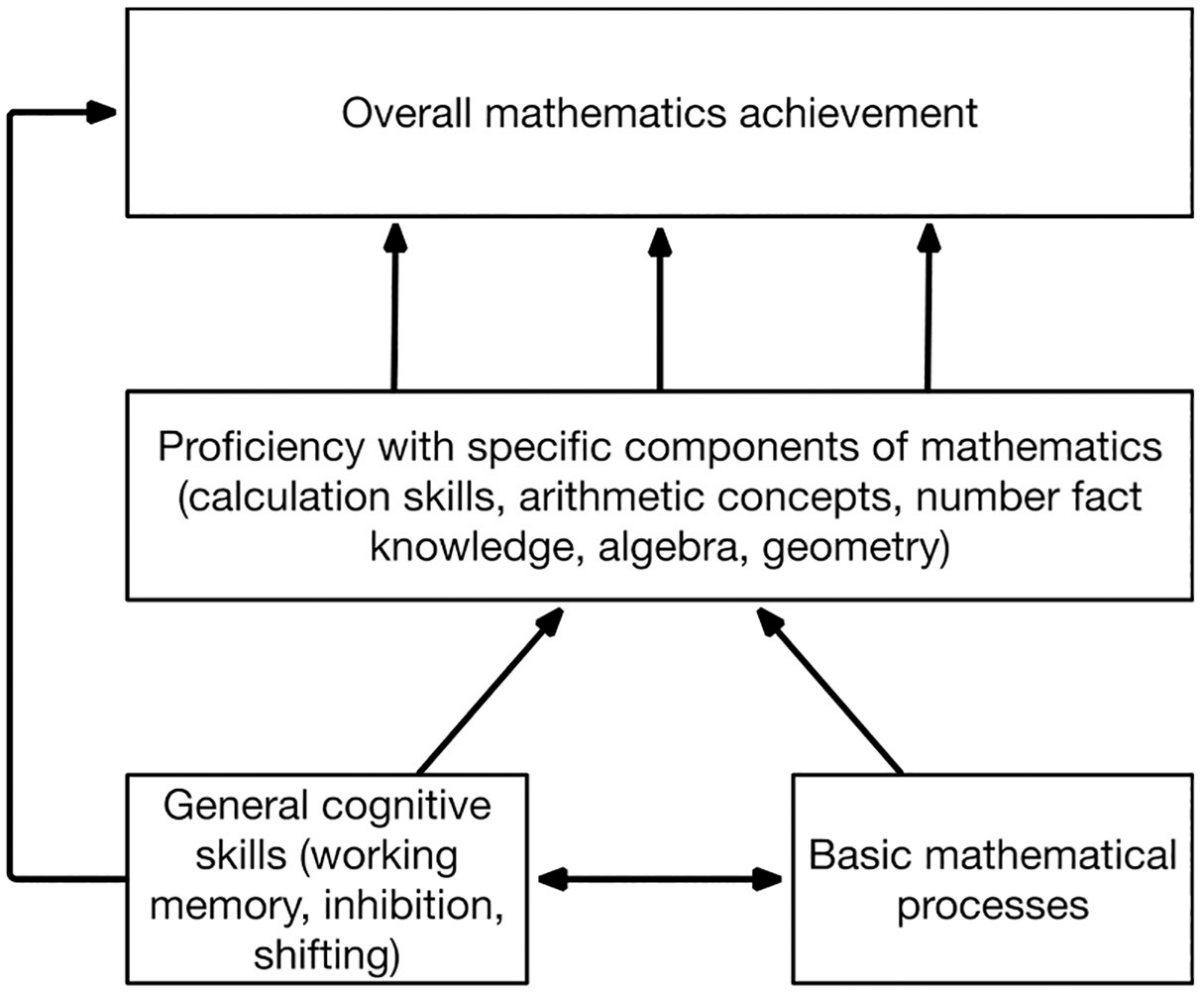
The multi-level framework of mathematics (adapted from Gilmore, 2023 [2]) indicating the specific components of mathematics and domain-general skills assessed in this study.

To understand the role of domain-general skills on mathematics it is crucial to consider the multi-componential nature of mathematics. Mathematics is not a unitary skill, but encompasses multiple specific skills and components of knowledge. The multi-level framework [2] (Fig 1) proposes that overall achievement in mathematics, i.e. as measured by curriculum assessments or broad standardised tests, arises out of proficiency with specific components of mathematics (e.g., calculation skills, number fact knowledge, algebra). These specific components in turn draw on basic mathematical processes such as symbolic comparison skills or numerical order processing. According to this framework, domain-general skills play a direct role in basic mathematical processes and specific components of mathematics [2, 3]. The relationship between domain-general skills and overall mathematical achievement may be fully explained by the role of domain-general skills in these specific components and basic processes, or domain-general skills may play an additional direct role in mathematics achievement. Here we investigate this framework with a broader range of specific components of mathematics skills than have been previously assessed.

Below we first consider the importance of EF skills in overall mathematical achievement (section 1.1) and arithmetic (1.2) before considering how these may be associated via direct and indirect relationships through specific components of mathematics (1.3). We then outline evidence about the relationship between executive function skills and algebra and geometry (1.4), and finally describe the current study (1.5).

### 1.1 EF skills and overall mathematical achievement

Throughout this paper, we use the term EF, which according to Miyake et al (2000)’s [3] model can be divided into three components. These are: updating or working memory (WM), the ability to hold information in mind and manipulate it; inhibition, the ability to suppress irrelevant or incorrect stimuli; and shifting, the ability to think flexibly in problem solving and shift attention between different stimuli.

Of the three components of EF, empirical evidence is strongest for the relationship between WM and mathematical achievement [4]. Syntheses of evidence show that both verbal and visuospatial WM are associated with mathematical achievement [4, 5], although the strength of the relationships with mathematical achievement may differ, with higher zero order correlations for measures of visuospatial WM (*r* = .60) and mathematics achievement, compared with verbal WM (*r* = .47) [6].

The roles of inhibition and shifting in mathematics achievement are less clear. In studies of adolescents and adults, inhibition tasks that involve non-numerical stimuli have a smaller or no significant association with mathematical achievement compared with inhibition tasks involving numerical stimuli. This was evidenced when the assessment of mathematics achievement focused on single and multi-digit arithmetic in children aged 6–8 years [7] or broader mathematical reasoning in individuals aged 8–25 years [8]. However, when included in a broader model of domain-general skills that also included visuospatial processing, non-numerical inhibition was an independent predictor of mathematical reasoning scores [9]. The literature is mixed as to whether inhibition is a predictor of mathematical achievement independent of WM [7, 10, 11] and shifting [10, 11]. These studies assessed mathematical achievement across a range of mathematical topics using the Group Mathematics Test [7], the Woodcock Johnson revised test (WJRT) [10] or the Cito mathematics test [11].

Moderate correlations between shifting and mathematical performance have been found in meta-analyses [4, 12] without controlling for WM, with some evidence that there are stronger correlations for younger children and those with mathematical difficulties [4]. However, shifting was not a predictor of mathematical achievement independent of WM and inhibition in another study [8]. Some of the mixed findings could be explained by age, as it is suggested that the involvement of inhibition and shifting may increase with increasing complexity of the mathematical tasks [13]. The majority of studies have focused on children in primary education. It is thus important to explore the role of these EF skills in mathematical achievement in secondary education where mathematical tasks become increasingly complex.

The mixed findings between EF component skills and mathematical achievement may be due to differences between tasks used across studies, especially for inhibition see [8]. EF tasks often draw upon multiple components of EF, such as when participants are required to hold a specific rule in WM when engaging in a switching task [14]. Therefore, different tasks may draw upon different constellations of skills. EF components are intercorrelated, so it is important to control for other components of EF when examining independent relationships between components of EF and mathematical achievement [3, 11].

### 1.2 EF skills and specific components of mathematics

The studies outlined above consider broad measures of overall mathematics achievement. However, to understand the role of executive function skills we must also consider the relationship with specific components of mathematics. The specific components of mathematics that have been the focus of previous research include calculation skills, number fact knowledge, word problem solving and understanding of arithmetic concepts. These different components of mathematics may draw on EF skills to different extents. For example, domain-general skills (which include measures of attention, language, phonological processing, processing speed, concept formation and WM) collectively explain more variance in procedural calculation skills compared with fact retrieval and conceptual understanding [15].

The specific relationships between individual EF skills (WM, inhibition, shifting) and individual components of mathematics may also differ, reflecting the different mechanisms involved. For example, WM may be used to hold interim solutions in mind during computation, problem representation and when accessing information stored in long-term memory [16]. Inhibition may be implicated through the suppression of unwanted number facts during retrieval. Finally, shifting may be involved through switching between different arithmetic procedures and representations of number, such as verbal numbers and written Arabic numerals [14, 17].

WM has been associated with number fact knowledge [8, 18, 19]. For example, measures of WM were associated with accuracy of addition and multiplication problems in children aged 6–8 years [18] and retrieval of addition and subtraction with totals less than 20 in children aged 7 to 9 years [19]. Cragg, Keeble, et al., 2017 measured fact retrieval using addition for primary-school aged participants, both addition and subtraction for secondary school-aged participants addition, subtraction, multiplication and division problems for undergraduate students (age range 8–25 years) and found associations with working memory in all age groups [8]. WM is also associated with calculation skills assessed using accuracy with written arithmetic in children aged 8–10 years [19] or assessed using the response time on mental arithmetic [8]. Conceptual understanding, assessed by asking the participant to identify and explain relationships between arithmetic problems was also associated with WM [8]. Mixed evidence has been found for an association between inhibition, shifting and number fact knowledge [8, 18].

### 1.3 Direct and indirect relationships between EF and mathematical achievement

In summary, evidence to date has demonstrated that EF skills (most strongly WM) are related to both overall mathematics achievement as well as some specific components of mathematics. This raises the question of whether and to what extent the relationship between EF skills and overall mathematical achievement can be explained by the role of these skills in specific components of mathematics. Previous research using cross-sectional data in children aged 8–10 years has identified that verbal working memory has a direct effect on arithmetic word problem solving, but no indirect effect via calculation skills. This study by Träff and colleagues used path analysis to test an a priori model where domain general skills are associated with one or more of symbolic number processing, number fact knowledge, procedural skills and arithmetic word problem solving [20]. This model also included measures of mental rotation, non-symbolic number comparison speed and language comprehension as additional variables [20]. Cragg, Keeble et al. (2017) also used cross-sectional data to examine direct and indirect effects of executive function skills [8]. Like Träff and colleagues, they found that in addition to an indirect association between executive function skills and mathematical achievement via specific components, a direct relationship between WM and mathematics achievement remained after controlling for the role of WM in number fact knowledge, procedural skills and conceptual understanding.

Why might a relationship between EF skills and mathematics achievement remain after controlling for the role of these skills in specific components of mathematics? It might be that EF skills are involved in more general processes such as problem representation and strategy selection [21]. These processes may be involved in problems that tap into ‘real-word’ use of mathematics that vary mathematical content across problems, typical of standardised assessments of mathematical achievement. Alternatively, EF skills might have a direct relationship with mathematics achievement over and above specific components because this reflects the role of EF skills in children’s learning in the classroom and their ability to integrate new knowledge with existing knowledge and skills. These roles for EF skills would not be captured by assessments of specific mathematical skills which typically involve repeated application of a particular procedures.

Alternatively, these studies may have found direct effects of executive function skills on mathematical achievement over and above the relationship with specific mathematics skills because they only consider a restricted set of components of mathematics. To date this research has only considered arithmetic-based specific mathematical skills [8]. However, there is a broader range of mathematics components, including geometry and algebra skills. These are a core part of the secondary school curriculum which are included in assessments of mathematical achievement at this level. Understanding how basic cognitive skills and specific components of mathematics combine and give rise to individual differences in overall mathematics achievement requires a broad perspective on mathematical skills beyond arithmetic. Below we consider the potential role of EF skills in algebra and geometry.

### 1.4 EF skills and algebra and geometry

#### 1.4.1 Algebra

Algebra is recognised as an important element of mathematics beyond arithmetic and is often considered a “gatekeeper” topic for more advanced mathematics and STEM subjects [22]. Students build their understanding of algebra on earlier arithmetical knowledge. For example, students transition from using predominantly concrete strategies to solve simple algebraic equations (e.g. inserting numbers) at age 13–14 years to more abstract rule-based strategies at older ages [23]. Success in algebra is dependent upon multiple skills, such as understanding of key concepts of pattern, function, equivalence and generalisation [24] and the correct application of procedural skills learnt using arithmetic such as relations, operations and their inverses [25]. However, if students have developed restricted conceptions of certain arithmetical principles (e.g. equivalence) this may need to be inhibited to allow appropriate use in algebra [26]. It is therefore possible that EF skills influence how easily students move from proficiency with arithmetic to understanding algebra.

There is some existing evidence that EF skills are associated with success in algebra. Specifically, studies have found an association between achievement in algebra and WM in students aged 11 and 15 years using verbal [27] or composite WM tasks [28]. Visuospatial but not verbal WM was significantly associated with performance on algebra and fraction tests in adolescents [29]. However, both verbal and visuospatial WM were associated with algebra performance in children aged 10 [30]. A meta-analysis including 27 correlations between algebra skills and WM found a small overall correlation (*r* = .27) [5].

Fewer studies have explored the association of other EF skills with algebra, yet it is plausible that the ability to shift attention and think flexibly could be associated with the accuracy of solving algebraic problems. However, no significant association was found between measures of inhibition and shifting and the ability to solve algebraic word problems in students aged 11 years [28]. When a latent measure of EF (with manifest variables of shifting and inhibition) was included in a model with WM and performance IQ, WM but not latent EF was significantly associated with performance on algebra word problems [31]. Further research is needed to identify the variance in algebra explained by different EF skills.

#### 1.4.2 Geometry

Geometry is related to higher order logical reasoning and judgement skills and thus may foster reasoning skills necessary for overall achievement in mathematics [32]. Again, there is less evidence regarding the involvement of EF skills in geometry compared with arithmetic. Visuospatial WM explained 20% of the variance in geometry skills, such as calculating the area of a figure [33]. Verbal WM, in addition to visuospatial WM, was associated with geometry skills in another study [34]. According to a meta-analysis, WM is significantly correlated with geometry, with no significant difference in the strength of the relationship between verbal and visuospatial WM [5]. However, the relationship between WM and geometry (*r* = .23) was weaker than the relationship between WM and whole number calculation (*r* = .35) or word-problem solving (*r* = .37) [5]. The relationship between WM and geometry appears to be stronger in younger, rather than older, individuals [5]. However, one recent study demonstrated that the ability to process spatial information, as opposed to visuospatial WM, is the unique predictor of geometry in children [35]. Given these inconsistencies, further research into the role of EF skills and the nature of the relationship between geometry and mathematical achievement is needed.

### 1.5 The Present study

This study sought to understand how EF skills, including WM, inhibition and shifting, are associated with specific components of mathematics as well as overall mathematical achievement. We extended the set of mathematical components investigated beyond arithmetic, typically the focus of research on this topic. We first tested the associations between measures of EF skills and calculation skills, number fact knowledge, understanding of arithmetic concepts, algebra and geometry in adolescents aged 12 to 14 years. Based on the existing literature, we hypothesised that WM would be significantly associated with each of these specific components of mathematics and also be directly associated with overall mathematical achievement. Given the mixed evidence for the association of inhibition and shifting with specific mathematical skills we did not specify directional hypotheses for these relationships. We hypothesised that each of the specific components of mathematics would be positively associated with overall mathematical achievement and would explain additional variance through this indirect relationship between EF skills and mathematics achievement. In doing so we aimed to further extend existing models of mathematics achievement [8, 36].

## 2. Method

### 2.1 Participants

The participants were 95 adolescents (48, 51% male) who attended mainstream secondary schools in the United Kingdom. Their mean age was 13.70 years (SD 0.74, range 11.82 to 15.10 years). Participants were spread across four academic years: Year 7 (6%), Year 8 (36%), Year 9 (45%) and Year 10 (13%). Pupils across all year groups had received instruction in algebra and geometry. The measures used were designed to capture variance in scores across these years. Participants comprised the control group for a study of mathematical skills in adolescents born <32 weeks of gestation. These between group comparisons have been published [37]. A sensitivity analysis using G*Power version 3.1.9.7 demonstrated that 95 participants gave 80% power to detect an effect size of f ^2^ = 0.14 in a multiple regression with 5 predictors and one covariate. Following the guidelines provided by Fritz and MacKinnon (2007) our sample is sufficient to identify indirect paths with combinations of medium and large effect sizes using a percentile bootstrap approach [38].

### 2.2 Tasks

Participants completed a battery of measures spread throughout a school day. Regular breaks were given. In addition to the measures listed below, participants completed additional tasks that were not related to the questions investigated here. The study received ethical approval from the Derbyshire NHS Research Ethics Committee (Ref 15/EM/0284). Parental consent and participant assent were obtained.

#### 2.2.1 Mathematics achievement

Mathematics achievement was assessed using the mathematical reasoning subtest of the Wechsler Individual Achievement Test 2nd UK edition (WIAT-II^UK^, [39]). Problems were context-based and were read aloud and presented visually. Participants could use paper for working but needed to give their answer verbally. The first 40 items from the starting point for this age group (item 21) included items on the following topics: arithmetic (9 items; mostly word problems), fractions (9), data handling (7), number patterns (5), time/date (4), geometry (4) and money (2). In this section of the WIAT there are no algebra items. Some problems included illustrations and many items involved multiple computational steps. Raw scores were used in the analysis.

#### 2.2.2 EF Skills

*2*.*2*.*2*.*1 Verbal WM*. A backwards word recall task was used to assess verbal WM. Participants had to verbally recall one syllable animal names in reverse order to that presented verbally by the experimenter. The list of animals increased from two to nine (4 trials of each span). Testing stopped when 3 trials in the same span were incorrect. Total correct number of trials were used.

*2*.*2*.*2*.*2 Visuospatial WM*. Visuospatial WM was assessed using the Mr X WM task from the standardized Automated Working Memory Assessment [40]. Participants were shown a series of pairs of rotated figures and asked to identify whether or not the figures were holding a ball in the same or different hand to one another. At the end of the sequence, they were asked to identify the locations of the ball held by one of the figures in the consecutive, correct order. Raw scores were used.

*2*.*2*.*2*.*3 Inhibition*. Inhibition was assessed using the inhibition subtest of the NEPSY-II [41]. Participants were asked to provide opposite names for shapes and arrows as quickly as possible (e.g. respond up for an arrow pointing down). The combined scaled score combines both accuracy and speed. The reliability score for the combined scaled score for age 13–16 was .73 as reported in the NEPSY-II manual.

*2*.*2*.*2*.*4 Shifting*. Shifting was assessed using the animal sorting subtest of the NEPSY-II. Participants were asked to sort eight animal cards into two categories with four cards in each. Participants had six minutes to identify as many categories as possible based on the characteristics of the card. The combined scaled score incorporates both the number of novel sorts and errors. The reliability score for the combined scaled score for age 13–16 was .96 as reported in the NEPSY-II manual.

#### 2.2.3 Specific Components of Mathematics

*2*.*2*.*3*.*1 Number Fact Knowledge*. Number fact knowledge was assessed using the age 13–14 version of the number fact knowledge task from Cragg, Keeble et al. (2017) [8]. Sixteen addition and subtraction problems (e.g. 11 + 4) were read aloud to participants. Participants were required to respond with the answer as quickly as possible without performing any mental calculation and to respond with ’I don’t know’ if they couldn’t recall the answer. The percentage of known number facts, i.e. facts recalled within three seconds, was recorded. McDonald’s Omega was .87 excluding trial 12 where there was no variance in the scores.

*2*.*2*.*3*.*2 Calculation skills*. Calculation skills were assessed using a composite of performance on mental and written arithmetic tasks. Mental calculation efficiency was assessed using the age 13–14 version of the procedural skills task from Cragg, Keeble et al. (2017) [8]. This task comprised 12 arithmetic problems presented on a computer screen and read aloud by the experimenter. Participants were instructed to solve the problem using any mental strategy they wished (retrieval, decomposition, counting, fingers). A verbal response was provided by participants and this answer and response time was recorded by the experimenter. The median response time for all correct trials for each participant was computed and standardised using z scores. Z scores were reversed by multiplying by -1 so that a higher z score indicated better performance.

Written calculation was assessed with a task based on the multi-digit arithmetic task used by Delazer et al. (2003) [42]. To avoid ceiling effects seven additional trials were added following pilot testing. Participants completed 16 problems on a worksheet (four each of addition, subtraction, multiplication and division), which were presented in a fixed order. Participants were asked to record their answer and working on the sheet. Addition and subtraction problems included two-, three- or four-digit numbers. Multiplication and division problems included one-, two- or three-digit numbers. The problems were presented horizontally on the worksheet, not in column format (e.g. 315 × 60 =). This task was untimed. Percentage accuracy was recorded and z scores computed. McDonald’s Omega was .80.

A composite calculation measure was calculated by summing the z scores across the mental and written arithmetic tests.

*2*.*2*.*3*.*3 Understanding of arithmetic concepts*. Conceptual understanding was assessed using the age 13–14 version of the conceptual knowledge task from Cragg, Keeble et al. (2017) [8]. A completed arithmetic problem with the answer was presented on a computer screen and read aloud by the researcher followed by a second problem with the answer missing. Participants were asked if they could use the first problem to help solve the second problem. They were not required to provide the answer, but to explain whether or not the first problem could be used to derive the answer to the second problem. Eighteen pairs of related trials, where the first problem could be used to solve the second problem, and twelve unrelated trial pairs were presented. The related trials were related by the principles of subtraction-complement principle (e.g. 148–73 = 75; 148–75 =), inverse operations (e.g. 15 × 6 = 90; 90 ÷6 =), and associative operations (e.g. 94–35–15 = 44; 94–50 =). The problems included addition and subtraction of two or three double-digit numbers, and multiplication and division of double-digit and single-digit numbers to prevent participants from attempting to solve them by computation. Out of the 12 unrelated trials, six were excluded as some participants identified alternative relationships. Twenty-four trials were included in the analysis. Percentage accuracy was recorded. McDonald’s Omega was .70.

*2*.*2*.*3*.*4 Algebra*. Algebra was assessed using 15 items (numbered 2a; 2b; 3; 4a; 4b; 4c; 5a; 5b; 5c; 6a; 11a; 11b; 16; 18b and 20) from the Concepts in Secondary Mathematics and Science algebra test [43]. The test was designed to evaluate pupils’ understanding and use of symbols in algebra, specifically to measure students reasoning about the use of letters in expressions (e.g. ignoring letters, evaluating letters, treating as a specific unknown, treating as a generalised unknown or treating as a variable) [44, 45]. The items involve comparing, manipulating and reasoning about algebraic expressions. This paper-based task had no time limit. Percentage accuracy was recorded. McDonald’s Omega was .79.

*2*.*2*.*3*.*5 Geometry*. Geometry was assessed using the first 15 items from the Van Hiele-Revised geometry test [46]. Items included identifying squares, triangles, rectangles, parallelograms and answering questions about the properties of squares, rectangles, rhombuses and intersecting circles. Questions were multiple choice from four options. This paper-based task had no time limit. Percentage accuracy was recorded. McDonald’s Omega was .52.

### 2.3. Data analysis

First, regression analyses were used to compare the variance in mathematical achievement explained by specific components of mathematics. Given that previous research has typically not included measures of algebra and geometry we were interested in identifying the variance in mathematical achievememnt explained when algebra and geometry were added to a model including calculation skills, number fact knowledge and understanding of arithmetic concepts. Second, regression analyses assessed the association between EF skills and overall mathematical achievement, number fact knowledge, calculation skills, conceptual understanding, algebra and geometry. Finally, mediation models were used to ascertain if the relationship between individual EF skills and overall mathematical achievement included indirect paths via the measures of specific mathematical skills, thereby explaining additional variance in mathematical achievement. All analyses were conducted in SPSS version 25. Age was included a covariate/predictor in all analyses.

## 3. Results

Descriptive statistics of mathematical achievement, EF and specific mathematical skills are presented in Table 1. The mean score on the conceptual knowledge task indicates ceiling effects may be present for this task.

**Table 1:** Descriptive statistics.

	N	Mean	SD	Minimum	Maximum	Skew	Kurtosis
Mathematical achievement (WIAT-II mathematical reasoning raw score)	95	53.80	5.78	40.0	66.0	-.19	-.45
EF skills							
Verbal WM (backwards word recall total correct) ^a^	94	14.55	4.49	6.0	27.0	.53	.29
Visuospatial WM (AWMA Mr X raw memory score) ^b^	94	16.25	5.63	6.0	34.0	.83	.49
Inhibition (NEPSY-II inhibition combined scaled score)	95	8.87	3.42	1.0	16.0	-.15	-.66
Shifting (NEPSY-II animal sorting combined scaled score)	95	9.95	3.42	1.0	17.0	-.06	-.51
Mathematical skills							
Number fact knowledge (% correct <3s)	95	77.43	20.97	27.0	100.0	-.54	-.94
Mental calculation skills (mental arithmetic median RT for all correct trials)	95	7.49	3.27	2.96	19.67	1.09	1.48
Written calculation skills (% correct on written arithmetic)	95	76.78	18.77	25.0	100.0	-.89	.31
Composite of calculation skills (z scores of reversed mental arithmetic RT + z scores of written arithmetic % correct)	95	0.0	1.69	-6.15	2.52	-.80	.62
Conceptual knowledge (% correct)	95	92.24	10.33	38.0	100.0	-2.53	8.29
Algebra (% correct)	95	66.11	19.39	7.0	100.0	-.37	-.16
Geometry (% correct)	95	48.98	15.0	13.0	87.0	.49	.08

^a^ One score missing due to experimenter error,

^b^ one score missing due to a technology issue

All EF and mathematical skills were significantly correlated with mathematical achievement (r = .27 to r = .73; Table 2) when age was controlled for.

**Table 2:** Correlations between EF, mathematical skills and mathematical achievement (n = 93).

Mathematical achievement	1	2	3	4	5	6	7	8	9	10	11	12
Mathematical achievement (1)	-	0.50**	0.55**	0.33**	0.27**	0.61**	0.55**	0.61**	0.70**	0.44**	0.73**	0.58**
Verbal WM (2)		-	0.30*	0.19	0.40**	0.25*	0.25*	0.30**	0.33**	0.19	0.45**	0.28**
Visuospatial WM (3)			-	0.07	0.14	0.45**	0.37**	0.37**	0.44**	0.32**	0.39**	0.37**
Inhibition (4)				-	0.24*	0.21*	0.21*	0.27**	0.29**	0.02	0.18	0.44**
Shifting (5)					-	0.29**	0.19	0.24*	0.26*	0.28**	0.23*	0.27*
Number fact knowledge (6)						-	0.64**	0.41**	0.63**	0.54**	0.47**	0.35**
Mental calculation skills (7)							-	0.40**	0.84**	0.59**	0.50**	0.32**
Written calculation skills (8)								-	0.83**	0.35**	0.51**	0.47**
Calculation skills composite (9)									-	0.57**	0.61**	0.47**
Conceptual skills (10)										-	0.51**	0.31**
Algebra (11)											-	0.52**
Geometry (12)												-
Age (13)	.07	.08	.14	.25*	- < .01	.11	- < .01	-.01	-.01	.01	.11	.14

Correlations are partial correlations controlling for age.

*p < .05,

**p < .01

### 3.1 Role of specific mathematical skills in mathematics achievement

A hierarchical regression analysis using age, number fact knowledge, composite calculation skills, and understanding of arithmetic concepts as predictors (model 1) explained 56% of the variance in mathematics achievement. Including algebra and geometry as additional predictors explained 71% of the variance, an additional 15% (Table 3). For model 2, number fact knowledge, composite calculation skills, algebra and geometry but not conceptual understanding were significant unique predictors of mathematics achievement. Age was not a significant predictor in the regression models. This suggests that the inter-individual variance in mathematical skill proficiency within ages masks any age differences across the sample.

**Table 3:** Hierarchical linear regression predicting mathematical achievement by specific mathematical skills.

	β Model 1	β Model 2
Age	.05	-.01
Number fact knowledge (%A)	.27**	.24**
Calculation skills (composite of z scores)	.56**	.31**
Conceptual understanding (%A)	-.01	-.12
Algebra (%A)	-	.42**
Geometry (%A)	-	.16*
R^2^	.56	.71
F Change	38.22**	22.17**

N = 95

**p < .01,

*p < .05

% A: percentage of items answered correctly, composite of z scores of reversed mental calculation reaction time and z scores of total correct items for written calculation

### 3.2 Role of EF skills in mathematics achievement and specific mathematical skills

Hierarchical regressions were conducted using age, verbal WM, visuospatial WM, inhibition and shifting as predictors of overall mathematics achievement, number fact knowledge, calculation skills, conceptual understanding, algebra and geometry (Table 4). Verbal WM was a significant independent predictor of overall mathematics achievement and algebra. Visuospatial WM was a significant independent predictor of overall mathematics achievement, number fact knowledge, calculation skills, conceptual understanding, algebra and geometry. Inhibition was a significant independent predictor of mathematics achievement, calculation skills and geometry. Shifting was a significant independent predictor of conceptual understanding only. The model including all the EF skills explained a significant amount of variance for overall mathematics achievement and for all the specific mathematical skills. EF skills explained the greatest variance in overall mathematics achievement (48%).

**Table 4:** Hierarchical linear regression predicting overall mathematical achievement and specific mathematical skills by EF skills.

	Mathematics achievement β	Number fact knowledge β	Calculation	Conceptual understanding β	Algebra β	Geometry β
Age	-.08	.02	-.12	-.02	.03	. < .01
Verbal WM (TC)	.325	.02	.14	.01	.33**	.07
Visuospatial WM (raw)	.446	.41**	.38**	.29**	.28**	.30**
Inhibition (SS)	.247	.14	.22**	-.07	.09	.38**
Shifting (SS)	.03	.19	.10	.25*	.04	.11
R^2^	.48	.28	.30	.16	.28	.34
F Change	20.34**	8.23**	9.08**	4.26**	8.42**	10.39**

**p < .01,

*p < .05

N = 93, missing data were excluded pairwise

TC- total items correct, raw = raw score, SS = Scaled score

### 3.3 Indirect relationships between EF skills and mathematics achievement

Finally, because three of the EF skills (verbal WM, visuospatial WM and inhibition) and four of the specific mathematical skills (number fact knowledge, calculation skills, geometry and algebra) were significant independent predictors of mathematics achievement, three separate mediation models were run (using the PROCESS macro, [47]) to test whether the relationship between each of the EF skills and overall mathematics achievement included significant indirect paths via the specific mathematical skills. Number fact knowledge, calculation skills, algebra and geometry were entered as mediators for each model. The dependent variable was overall mathematics achievement. Age was entered as a covariate in all models (Table 5).

**Table 5:** Age added as a covariate for all models. Parallel mediation analysis between predictors visuospatial WM, verbal WM and inhibition, with mathematics achievement as the outcome and mathematical component skills as mediators.

	Predictor	Completely standardised point estimate ^a^^b^	SE ^b^	p	95% Confidence Interval ^b^^c^		R^2^
					Lower interval	Upper interval	
Domain specific mediators of the relationship between visuospatial WM and mathematics achievement (n = 94)							
Total effect (c) (X to Y)	Visuospatial WM	.55	.09	< .001	.38	.73	.31
Direct effect (c’) (X to Y controlling for M^1^, M^2^, M^3^, M^4^)	Visuospatial WM	.18	.07	.012	.04	.31	.71
Indirect effects							
Total		.38	.06		.25	.49	
Number fact knowledge a_1_b_1_*		.08	04		< .01	.17	
Calculation skills a_2_b_2_*		.11	.04		.04	.20	
Algebra a_4_b_4_*		.14	.04		.06	.22	
Geometry a_5_b_5_*		.06	.03		.01	.12	
Domain specific mediators of the relationship between verbal WM and mathematics achievement (n = 94)							
Total effect (c) (X to Y)	Verbal WM	.52	.09	< .001	.34	.70	.27
Direct effect (c’) (X to Y controlling for M^1^, M^2^, M^3^, M^4^)	Verbal WM	.18	.06	.005	.06	.31	.73
Indirect effects							
Total		.34	.06		.21	.46	
Number fact knowledge a_1_b_1_*		.05	.03		< .01	.11	
Calculation skills a_2_b_2_*		.09	.04		.03	.17	
Algebra a_4_b_4_*		.16	.04		.08	.24	
Geometry a_5_b_5_*		.05	.03		< .01	.10	
Domain specific mediators of the relationship between inhibition and mathematics achievement (n = 95)							
Total effect (c) (X to Y)	Inhibition	.35	.10	.001	.15	.55	.12
Direct effect (c’) (X to Y controlling for M^1^, M^2^, M^3^, M^4^)	Inhibition	.09	.07	.173	-.04	.23	.71
Indirect effects							
Total		.25	.09		.08	.43	
Number fact knowledge a_1_b_1_*		.05	.03		< .01	.12	
Calculations skills a_2_b_2_*		.08	.04		.02	.18	
Algebra a_4_b_4_		.07	.05		-.02	.17	
Geometry a_5_b_5_		.05	.04		-.02	.12	

*Significant independent mediator as confidence interval does not straddle zero.

^a^ Completely standardised point estimate calculated according to the following formula $\frac{{SD}_{X}(z)}{{SD}_{Y}}$ z = effect coefficient (e.g. a^1^b^2^, c). Used PROCESS generated completed standardised estimates and SE for all indirect effects.

^b^ Completely standardised point estimate, SE and confidence interval calculated manually for total effect (c) and direct effect (c’).

^c^ Bootstrapped confidence intervals for indirect effects.

**Fig 2:**
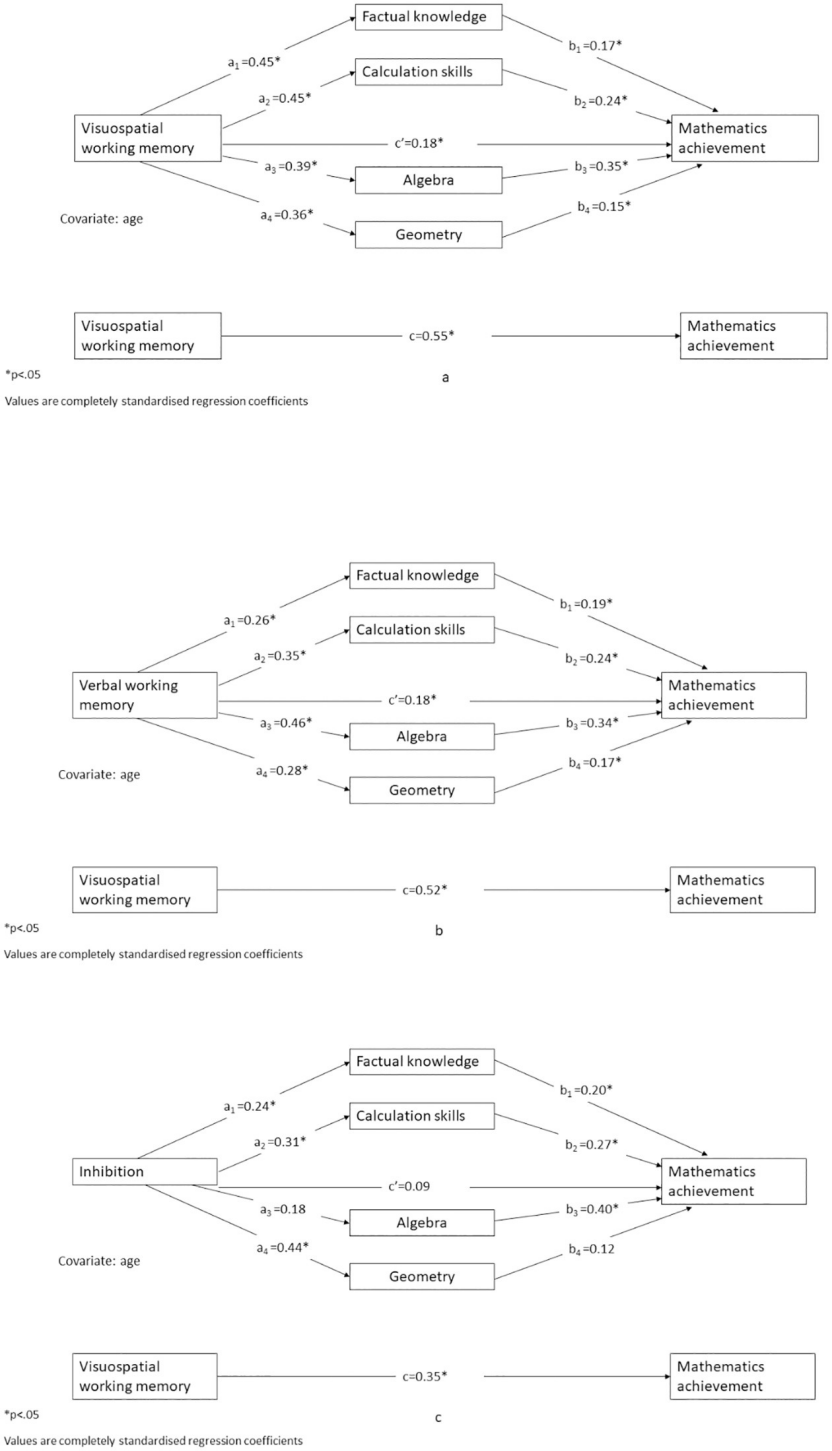
a. Mediation model between visuospatial working memory, mathematical component skills and mathematics achievement. b. Mediation model between verbal working memory, mathematical component skills and mathematics achievement. c. Mediation model between inhibition, mathematical component skills and mathematics achievement.

For visuospatial WM, there were significant indirect effects on mathematics achievement through all four specific mathematical skills (number fact knowledge, calculation skills, algebra and geometry). The indirect path via algebra was the strongest. There remained a significant direct effect of visuospatial WM on mathematics achievement once the mediators were controlled for (see Fig 2a).

For verbal WM, there were significant indirect effects on mathematics achievement through all four specific mathematical skills. The indirect path via algebra was again the strongest. A significant direct effect of verbal WM on mathematics achievement remained after controlling for the mediators (see Fig 2b).

For inhibition, there were significant indirect effects on mathematics achievement through number fact knowledge and calculation skills. The indirect paths through algebra and geometry were not significant. After controlling for the mediators, there was no longer a direct effect of inhibition on mathematics achievement (see Fig 2c).

## 4. Discussion

Our results confirm and extend existing models of mathematics [8, 36] through the inclusion of a broader range of specific mathematical skills including algebra and geometry. In support of our hypothesis, we found that both visuospatial and verbal WM was associated with number fact knowledge, calculation skills, algebra and geometry performance Only visuospatial WM was associated with understanding of arithmetic concepts. We found that visuospatial WM, verbal WM and inhibition were indirectly associated with mathematics achievement via specific mathematical component skills. Our findings provide further evidence for relationships between specific EF and specific mathematical skills, which are in turn associated with overall mathematics achievement. In other words, the well-established association between EF skills and mathematics achievement is somewhat, in the case of WM, or substantially, in the case of inhibition, explained by the association between these EF skills and specific components of mathematics. Our findings shed light on the role of EF skills in mathematics achievement, taking a multi-componential view of the nature of mathematics, with implications for understanding the challenges of learning mathematics. Below we first consider the importance of different components of mathematics for overall achievement in mathematics and the role that EF skills may play in each of these components before discussing the mechanisms which may underpin the direct and indirect relationships that exist between these skills.

### 4.1 Relationships with mathematics achievement

#### 4.1.1 Algebra and geometry

Our study included a broader range of specific mathematical skills than most previous research. The inclusion of algebra and geometry tasks explained an additional 15% of variance in mathematical achievement compared with that explained by number fact knowledge, conceptual understanding of arithmetic, and calculation skills. This demonstrates the importance of broad assessments of the mathematics curriculum to understand what contributes to individual differences in mathematical outcomes. Our finding that algebra performance was the strongest predictor of WIAT mathematics, despite this measure including no algebra items, achievement measure supports the hypothesis that algebra is crucial component of mathematics achievement. However, it is important to note that our findings are correlational and so it is possible that proficiency with a broad measure of mathematics achievement may also predict competency in algebra. Therefore, further longitudinal research is needed to understand the directional relationship between algebra and mathematics achievement. Compared with algebra, geometry performance explained less variance in mathematics achievement. It has been shown that geometry and mathematical reasoning share higher order logical reasoning and judgement skills [32]. Therefore, like for algebra, the direction of the relationship with overall achievement is unclear.

#### 4.1.2 Arithmetic skills

It is important to note that, even in secondary school, basic arithmetic skills (calculation skills, number fact knowledge and understanding of arithmetic concepts) were significantly associated with mathematics achievement, explaining 56% of the variance. Previous research has shown that arithmetic skills are foundational for later mathematics achievement: arithmetic proficiency assessed at age 8 was an independent predictor of mathematics achievement assessed at ages 11 and 14 [48] and poor foundational skills can result in a persistent gap in achievement [49]. This highlights the importance of consolidating basic arithmetic skills throughout primary and into secondary school.

Basic arithmetic skills comprise knowledge of number facts, the ability to carry out calculation procedures, and understanding of arithmetical concepts. Consistent with the findings of Cragg, Keeble et al. (2017) factual knowledge and calculation skills each explained unique variance in mathematical achievement. However, in contrast to Cragg, Keeble et al. (2017) understanding of arithmetic concepts was not a significant independent predictor of mathematics achievement in the current study. Previous research has highlighted the importance of conceptual knowledge for success in mathematics, and procedural and conceptual skills are mutually co-dependent [50]. In line with this we found strong correlations between understanding of arithmetic concepts, factual knowledge and calculation skills in the present study (see Table 2). It is possible that the lack of a unique relationship between conceptual understanding and mathematics achievement in our study was because our conceptual measure failed to provide a valid measure of students’ understanding in this sample due to ceiling effects. The mean accuracy in the present sample was 92% compared with mean accuracy of 76–81% for 11 to 14-year-olds in Cragg, Keeble et al.’s (2017) study.

#### 4.1.3 EF skills

Commensurate with previous research, verbal WM, visuospatial WM and inhibition were associated with overall mathematics achievement [4]. Shifting did not significantly independently contribute towards mathematics achievement and it was only associated with conceptual understanding. This aligns with mixed evidence for the role of shifting in mathematics achievement [4, 7, 10, 11]. It is important that studies include tasks which measure all components of EF, to enable the comparison of their relative predictive power. This is illustrated in the present study, as whilst the correlations with mathematics achievement were of comparable size for inhibition and shifting, inhibition remained a unique predictor of mathematics achievement independent of other components of EF, whereas shifting did not.

### 4.2 Relationships between EF and mathematical component skills

Few studies have considered the role of EF skills beyond components of arithmetic. We found that EF skills explained similar proportions of variance in geometry (34%) and algebra (28%) performance. In accordance with previous studies, we found a relationship between algebra performance and both visuospatial and verbal WM [29, 30] but not inhibition or shifting [28, 30]. These findings suggest that solving simple algebraic equations might draw upon both verbal and visuospatial resources, for example items such as "What can you say about m if m = 3n +1 and n = 4?" might draw on visuo-spatial strategies to manipulate the structure of the problem and verbal strategies to retrieve number facts from long term memory and hold interim solutions in mind.

Visuospatial WM, but not verbal WM was associated with geometry performance. This is to be expected given that identifying and evaluating the properties of shapes draws upon visuospatial processes. The role of inhibition in geometry to our knowledge has not been previously studied, but we found that inhibition was significantly associated with geometry performance. However, this association may have arisen due to the visuospatial processing required in the inhibition task by responding to shapes or the direction of an arrow. EF skills cannot be measured independently of the specific content (e.g. verbal or visuospatial information) to be processed. Using alternative measures of inhibition skills, or a latent factor across multiple measures of this construct would demonstrate if inhibition skills are indeed associated with geometry performance independently of visuospatial processing.

Our measures of mathematics skills were assessed at a single time-point. Therefore, we cannot distinguish between the executive function skills involved in the process of learning mathematics with those involved in mathematics performance. Other research is beginning to unpick these different roles for executive functions and has found different roles in learning vs. task performance [51]. Further longitudinal research that is embedded in learning contexts is required to further understanding these different roles.

### 4.3 Direct and indirect pathways of mathematics achievement

One of the main aims of the current study was to replicate and extend models of mathematics achievement [2, 8, 36]. We found that both verbal and visuospatial WM were indirectly associated with mathematics achievement via factual knowledge, calculation skills, algebra and geometry performance. There are two potential mechanisms via which these indirect relationships may operate. First, EF skills may be actively required for performing specific mathematical processes and mathematical achievement is subsequently built upon these specific skills. For example, WM may be required to retrieve number facts from long term memory, hold interim solutions in mind while performing arithmetic operations, rearrange algebraic equations and manipulate spatial representations. Secondly, EF skills may also be foundational for learning mathematical skills in the classroom. To learn and understand mathematical ideas and procedures children need to process a large amount of new material. Therefore, children with lower WM capacity may develop gaps in their understanding of concepts, procedures, and number facts and this may impact on overall mathematics achievement. Studies have shown that WM capacity is not only associated with performance of mathematics but also growth in mathematics over time [52]. These two mechanisms are not mutually exclusive, and both may contribute to the relationship between WM and mathematics achievement.

Consistent with previous findings [53], a direct association between WM (verbal and visuospatial) and mathematics achievement remained after accounting for the indirect paths. Therefore, WM plays an additional role in measures of mathematics achievement that is not captured by measures of specific skills. WM may be involved in more general processes of problem representation and strategy selection that are particularly important in context-rich or word problems [8, 21] or this may reflect the role of WM in classroom learning and children’s ability to combine new knowledge with existing knowledge and skills. Use of contextually-rich problems when assessing mathematics achievement provides valuable insight into the skills involved, as this reflects the application of mathematics to real life, and skills and knowledge important to further education in STEM [54].

In contrast to WM, we found that inhibition was only indirectly associated with mathematics achievement via number fact knowledge and calculation skills. This is in line with previous correlational studies [8], as well as evidence from experimental studies showing that children with poorer number fact knowledge were more sensitive to interference [55]. It is possible that children who are less able to inhibit prepotent responses may struggle to retrieve facts quickly due to interference from associative neighbouring facts [56]. For calculation skills, inhibition may be required to suppress the use of a previously learnt but now superseded strategy, such as decomposition as opposed to retrieval [57]. The present study found that inhibition was not associated with mathematics achievement over and above its involvement in specific arithmetic skills.

### 4.4 Limitations

A limitation to the present study is the lack of confirmatory factor analysis for a three-factor model of executive functioning. As this study included an extensive battery of measures as part of a larger study of numerical processing following very preterm birth, it was not possible to include additional tasks necessary to create a latent measure of each EF component. Nevertheless, the moderate correlations among the EF skills and the differential relationships we found between components of EF and mathematics achievement provide support for three dissociable components [58]. Similarly, due to the breadth of the mathematical skills we wished to include we were only able to include a single measure per component (e.g. calculation, algebra) and therefore we cannot distinguish between the task measure and the underlying construct. Future research should incorporate multiple measures per construct and so the skills specific to the measure and those specific to the construct can be distinguished.

According to published guidance [38], our sample was sufficient to run hierarchical regression models and to investigate indirect effects. Although, the size of our sample was small for the complexity of our analyses. However, our analyses were theoretically-driven and replicated (and extended) previous findings which we believe adds weight to these findings. Future research should replicate these findings in a larger sample.

In addition, these data were cross-sectional, and limit the interpretation of causality. Whilst we acknowledge that there are concerns with inferring the temporal ordering of variables in the mediation model from cross-sectional data, there is substantial existing evidence for longitudinal relationships between EF skills and specific mathematical skills, between EF skills and overall mathematics achievement, and between specific mathematical skills and mathematics achievement. For example, it has been shown that teacher-rated EF is a longitudinal predictor of mathematics grade point average in adolescents [59]. Similarly, proficiency in specific arithmetic skills is a longitudinal predictor of mathematics achievement several years later [60, 61]. Whilst there may be a bidirectional relationship between executive function skills and mathematics achievement from preschool to kindergarten [62] and from kindergarten to second grade in the USA [63], there is a paucity of research on whether mathematical skills longitudinally predict executive function skills during adolescence. Further studies with multiple assessment time points should investigate whether bidirectionality between mathematics achievement and executive function skills is evidenced using cross-lagged models across years of secondary education.

### 4.5 Conclusion

In conclusion, algebra and geometry performance are important predictors of mathematics achievement, each accounting for unique variance in mathematics achievement in secondary school pupils. We replicated existing models of mathematics skills in which the role of WM and inhibition are indirectly related to mathematics achievement via arithmetic factual knowledge and procedural skills [8] as well as extending this model to include algebra and geometry performance as alternative indirect pathways. Studies that include both performance and learning measures of specific mathematical processes as well as context-rich assessments of mathematics achievement and mathematical problem solving are needed to differentiate the multiple mechanisms by which EF skills may be involved in mathematics outcomes.

## References

[1] Parsons S, Bynner J. Does numeracy matter more?: National Research and Development Centre for adult literacy and numeracy.; 205.

[2] Gilmore C. EXPRESS: Understanding the complexities of mathematical cognition: A multi-level framework. Quarterly Journal of Experimental Psychology. 2023;0(ja):17470218231175325. 10.1177/17470218231175325 PMC10466984 37129432

[3] Miyake A, Friedman NP, Emerson MJ, Witzki AH, Howerter A, Wager TD. The unity and diversity of executive functions and their contributions to complex “frontal lobe” tasks: A latent variable analysis. Cognitive psychology. 2000;41(1):49–100. doi: 10.1006/cogp.1999.0734 10945922

[4] Friso-Van den Bos I, Van der Ven SH, Kroesbergen EH, Van Luit JE. Working memory and mathematics in primary school children: A meta-analysis. Educational research review. 2013;10:29–44.

[5] Peng P, Namkung J, Barnes M, Sun C. A meta-analysis of mathematics and working memory: Moderating effects of working memory domain, type of mathematics skill, and sample characteristics. Journal of Educational Psychology. 2016;108(4):455.

[6] Szűcs D, Devine A, Soltesz F, Nobes A, Gabriel F. Cognitive components of a mathematical processing network in 9‐year‐old children. Developmental science. 2014;17(4):506–24. doi: 10.1111/desc.12144 25089322 PMC4253132

[7] Bull R, Scerif G. Executive functioning as a predictor of children’s mathematics ability: Inhibition, switching, and working memory. Developmental neuropsychology. 2001;19(3):273–93. doi: 10.1207/S15326942DN1903_3 11758669

[8] Cragg L, Keeble S, Richardson S, Roome HE, Gilmore C. Direct and indirect influences of executive functions on mathematics achievement. Cognition. 2017;162:12–26. doi: 10.1016/j.cognition.2017.01.014 28189034

[9] Gilmore C, Clayton S, Cragg L, McKeaveney C, Simms V, Johnson S. Understanding arithmetic concepts: The role of domain-specific and domain-general skills. PloS one. 2018;13(9):e0201724. doi: 10.1371/journal.pone.0201724 30252852 PMC6155447

[10] Espy KA, McDiarmid MM, Cwik MF, Stalets MM, Hamby A, Senn TE. The contribution of executive functions to emergent mathematic skills in preschool children. Developmental neuropsychology. 2004;26(1):465–86. doi: 10.1207/s15326942dn2601_6 15276905

[11] Van der Ven SH, Kroesbergen EH, Boom J, Leseman PPM. The development of executive functions and early mathematics: A dynamic relationship. British Journal of Educational Psychology. 2012;82(1):100–19. doi: 10.1111/j.2044-8279.2011.02035.x 22429060

[12] Yeniad N, Malda M, Mesman J, Van IJzendoorn MH, Pieper S. Shifting ability predicts math and reading performance in children: A meta-analytical study. Learning and Individual Differences. 2013;23:1–9.

[13] Toll SWM, Van der Ven SH, Kroesbergen EH, Van Luit JE. Executive functions as predictors of math learning disabilities. Journal of Learning Disabilities. 2011;44(6):521–32. doi: 10.1177/0022219410387302 21177978

[14] Bull R, Lee K. Executive functioning and mathematics achievement. Child Development Perspectives. 2014;8(1):36–41.

[15] Fuchs LS, Compton DL, Fuchs D, Paulsen K, Bryant JD, Hamlett CL. The prevention, identification, and cognitive determinants of math difficulty. Journal of educational psychology. 2005;97(3):493.

[16] Gilmore C, Keeble S, Richardson S, Cragg L. The interaction of procedural skill, conceptual understanding and working memory in early mathematics achievement. Journal of Numerical Cognition. 2017;3(2).

[17] Cragg L, Gilmore C. Skills underlying mathematics: The role of executive function in the development of mathematics proficiency. Trends in neuroscience and education. 2014;3(2):63–8.

[18] Bellon E, Fias W, De Smedt B. More than number sense: The additional role of executive functions and metacognition in arithmetic. Journal of experimental child psychology. 2019;182:38–60. doi: 10.1016/j.jecp.2019.01.012 30807905

[19] Cowan R, Powell D. The contributions of domain-general and numerical factors to third-grade arithmetic skills and mathematical learning disability. Journal of educational psychology. 2014;106(1):214. doi: 10.1037/a0034097 24532854 PMC3906804

[20] Traff U, Olsson L, Skagerlund K, Ostergren R. Cognitive mechanisms underlying third graders’ arithmetic skills: Expanding the pathways to mathematics model. Journal of Experimental Child Psychology. 2018;167:369–87. doi: 10.1016/j.jecp.2017.11.010 29232622

[21] Viterbori P, Traverso L, Usai MC. The role of executive function in arithmetic problem-solving processes: A study of third graders. Journal of Cognition and Development. 2017;18(5):595–616.

[22] National Mathematics Advisory Panel. Foundations for success: The final report of the National Mathematics Advisory Panel. US Department of Education; 2008.

[23] Susac A., Bubic A., Vrbanc A., & Planinic. Development of abstract mathematical reasoning: the case of algebra. Frontiers in Human Neuroscience. 2014 8, 679. doi: 10.3389/fnhum.2014.00679 25228874 PMC4151197

[24] Day L, Stephens M, Horne M. Developing learning progressions to support mathematical reasoning in the middle years-algebraic reasoning. MERGA. 2017;40.

[25] Kieran C. Algebraic thinking in the early grades: What is it. The mathematics educator. 2004;8(1):139–51.

[26] McNeil NM, Alibali MW. Why won’t you change your mind? Knowledge of operational patterns hinders learning and performance on equations. Child development. 2005;76(4):883–99. doi: 10.1111/j.1467-8624.2005.00884.x 16026503

[27] Geary DC, Hoard MK, Nugent L, Rouder JN. Individual differences in algebraic cognition: Relation to the approximate number and semantic memory systems. Journal of Experimental Child Psychology. 2015;140:211–27. doi: 10.1016/j.jecp.2015.07.010 26255604 PMC4558338

[28] Lee K, Ng EL, Ng SF. The contributions of working memory and executive functioning to problem representation and solution generation in algebraic word problems. Journal of Educational Psychology. 2009;101(2):373.

[29] Abreu-Mendoza RA, Chamorro Y, Garcia-Barrera MA, Matute E. The contributions of executive functions to mathematical learning difficulties and mathematical talent during adolescence. PLoS One. 2018;13(12):e0209267. doi: 10.1371/journal.pone.0209267 30543713 PMC6292664

[30] Lee K, Ng SF, Bull R, Pe ML, Ho RHM. Are patterns important? An investigation of the relationships between proficiencies in patterns, computation, executive functioning, and algebraic word problems. Journal of Educational Psychology. 2011;103(2):269.

[31] Lee K, Ng SF, Bull R. Learning and solving algebra word problems: The roles of relational skills, arithmetic, and executive functioning. Developmental psychology. 2018;54(9):1758. doi: 10.1037/dev0000561 30148402

[32] Tatsuoka KK, Corter JE, Tatsuoka C. Patterns of diagnosed mathematical content and process skills in TIMSS-R across a sample of 20 countries. American educational research journal. 2004;41(4):901–26.

[33] Kyttälä M, Lehto JE. Some factors underlying mathematical performance: The role of visuospatial working memory and non-verbal intelligence. European Journal of Psychology of Education. 2008;23(1):77–94.

[34] Giofrè D, Mammarella IC, Cornoldi C. The relationship among geometry, working memory, and intelligence in children. Journal of Experimental Child Psychology. 2014;123:112–28. doi: 10.1016/j.jecp.2014.01.002 24709286

[35] Hawes Z, Moss J, Caswell B, Seo J, Ansari D. Relations between numerical, spatial, and executive function skills and mathematics achievement: A latent-variable approach. Cognitive Psychology. 2019;109:68–90. doi: 10.1016/j.cogpsych.2018.12.002 30616227

[36] Geary DC. Mathematics and learning disabilities. Journal of learning disabilities. 2004;37(1):4–15. doi: 10.1177/00222194040370010201 15493463

[37] Clayton S, Simms V, Cragg L, Gilmore C, Marlow N, Spong R, et al. Etiology of persistent mathematics difficulties from childhood to adolescence following very preterm birth. Child Neuropsychology. 2022;28(1):82–98. doi: 10.1080/09297049.2021.1955847 34472423

[38] Fritz MS, MacKinnon DP. Required sample size to detect the mediated effect. Psychological science. 2007;18(3):233–9. doi: 10.1111/j.1467-9280.2007.01882.x 17444920 PMC2843527

[39] Wechsler WD. Wechsler Individual Achievement Test. 2nd edition ed: Pearson; 2004.

[40] Alloway TP, Gathercole SE, Kirkwood H, Elliott J. Evaluating the validity of the automated working memory assessment. Educational Psychology. 2008;28(7):725–34.

[41] Korkman M, Kirk U, Kemp S. NEPSY II: Clinical and interpretive manual: Harcourt Assessment, PsychCorp; 2007.

[42] Delazer M, Girelli L, Granà A, Domahs F. Number processing and calculation–normative data from healthy adults. The clinical neuropsychologist. 2003;17(3):331–50. doi: 10.1076/clin.17.3.331.18092 14704884

[43] Hodgen J, Brown M, Kuchemann D. English school students’ understanding of algebra, in the 1970s and now. Der Mathematikunterricht. 2010.

[44] Bisson M-J, Gilmore C, Inglis M, Jones I. Measuring conceptual understanding using comparative judgement. International Journal of Research in Undergraduate Mathematics Education. 2016;2(2):141–64.

[45] Hodgen J, Kuchemann D, Brown M. Learning experiences designed to develop algebraic thinking: Lessons from the ICCAMS project in England. Learning experiences to promote mathematics learning: Yearbook 2014 Association of Mathematics Educators: World Scientific; 2014. p. 171–86.

[46] Usiskin Z. Van Hiele Levels and Achievement in Secondary School Geometry. CDASSG Project. 1982.

[47] Hayes AF. Introduction to mediation, moderation, and conditional process analysis: A regression-based approach: Guilford publications; 2017.

[48] Nunes T, Bryant P, Barros R, Sylva K. The relative importance of two different mathematical abilities to mathematical achievement. British Journal of Educational Psychology. 2012;82(1):136–56. doi: 10.1111/j.2044-8279.2011.02033.x 22429062

[49] Morgan PL, Farkas G, Wu Q. Five-year growth trajectories of kindergarten children with learning difficulties in mathematics. Journal of Learning Disabilities. 2009;42(4):306–21. doi: 10.1177/0022219408331037 19299551

[50] Rittle-Johnson B, Schneider M, Star JR. Not a one-way street: Bidirectional relations between procedural and conceptual knowledge of mathematics. Educational Psychology Review. 2015;27(4):587–97.

[51] Gilmore C, Simsek, E., Eaves, J., & Cragg L. The role of cognitive and applied executive function skills in learning new mathematics material.. Under Review.

[52] Geary DC, Hoard MK, Nugent L, Bailey DH. Mathematical cognition deficits in children with learning disabilities and persistent low achievement: a five-year prospective study. Journal of educational psychology. 2012;104(1):206. doi: 10.1037/a0025398 27158154 PMC4855881

[53] Cragg L, Richardson S, Hubber PJ, Keeble S, Gilmore C. When is working memory important for arithmetic? The impact of strategy and age. PloS one. 2017;12(12):e0188693. doi: 10.1371/journal.pone.0188693 29228008 PMC5724815

[54] Fuchs L, Fuchs D, Seethaler PM, Barnes MA. Addressing the role of working memory in mathematical word-problem solving when designing intervention for struggling learners. ZDM. 2020;52(1):87–96.

[55] De Visscher A, Noël MP. Arithmetic facts storage deficit: The hypersensitivity‐to‐interference in memory hypothesis. Developmental science. 2014;17(3):434–42. doi: 10.1111/desc.12135 24410798

[56] LeFevre J-A, Kulak AG, Bisanz J. Individual differences and developmental change in the associative relations among numbers. Journal of Experimental Child Psychology. 1991;52(2):256–74.

[57] Mammarella IC, Caviola S, Giofrè D, Borella E. Separating math from anxiety: The role of inhibitory mechanisms. Applied Neuropsychology: Child. 2018;7(4):342–53. doi: 10.1080/21622965.2017.1341836 28682117

[58] Lee K, Bull R, Ho RM. Developmental changes in executive functioning. Child development. 2013;84(6):1933–53. doi: 10.1111/cdev.12096 23550969

[59] Samuels WE, Tournaki N, Blackman S, Zilinski C. Executive functioning predicts academic achievement in middle school: A four-year longitudinal study. The Journal of Educational Research. 2016;109(5):478–90.

[60] Casey BM, Pezaris E, Fineman B, Pollock A, Demers L, Dearing E. A longitudinal analysis of early spatial skills compared to arithmetic and verbal skills as predictors of fifth-grade girls’ math reasoning. Learning and Individual Differences. 2015;40:90–100.

[61] Geary DC, Nicholas A, Li Y, Sun J. Developmental change in the influence of domain-general abilities and domain-specific knowledge on mathematics achievement: An eight-year longitudinal study. Journal of Educational Psychology. 2017;109(5):680. doi: 10.1037/edu0000159 28781382 PMC5542417

[62] Schmitt SA, Geldhof GJ, Purpura DJ, Duncan R, McClelland MM. Examining the relations between executive function, math, and literacy during the transition to kindergarten: A multi-analytic approach. Journal of Educational Psychology. 2017;109(8):1120.

[63] Miller-Cotto D, Byrnes JP. What’s the best way to characterize the relationship between working memory and achievement?: An initial examination of competing theories. Journal of Educational Psychology. 2020;112(5):1074.

